# The impact of clinical pharmacist services on patient health outcomes in Pakistan: a systematic review

**DOI:** 10.1186/s12913-021-06897-0

**Published:** 2021-08-23

**Authors:** Ali Ahmed, Muhammad Saqlain, Maria Tanveer, Ali Qais Blebil, Juman Abdulelah Dujaili, Syed Shahzad Hasan

**Affiliations:** 1 School of Pharmacy, Monash University, Jalan Lagoon Selatan, Bandar Sunway, 47500 Subang Jaya, Selangor Malaysia; 2 Department of Pharmacy, Quaid I Azam University, Islamabad, Pakistan; 3 School of Applied Sciences, Department of Pharmacy, University of Huddersfield, Huddersfield, UK

**Keywords:** Low-and middle-income countries, Pharmacoeconomics, Pharmaceutical care, Therapeutics, Humanistic, Clinical

## Abstract

**Background:**

The pharmacist’s role shifts from dispensing to bedside care, resulting in better patient health outcomes. Pharmacists in developed countries ensure rational drug use, improve clinical outcomes, and promote health status by working as part of a multidisciplinary team of healthcare professionals. However, clinical pharmacist services on healthcare utilization in low-and middle-income countries (LMICs) like Pakistan are unclear. As a result, we aim to systematically review pharmacists’ clinical roles in improving Pakistani patients’ therapeutic, safety, humanistic, and economic outcomes.

**Methods:**

We searched PubMed, Scopus, EMBASE, CINAHL, and Cochrane Library for relevant articles published from inception to 28th February 2021. All authors were involved in the screening and selection of studies. Original studies investigating the therapeutic, humanistic, safety, and economic impact of clinical pharmacists in Pakistani patients (hospitalised or outpatients) were selected. Two reviewers independently assessed the risk of bias in studies, and discrepancies were resolved through mutual consensus. All of the included studies were descriptively synthesised, and PRISMA reporting guidelines were followed.

**Results:**

The literature search found 751 articles from which nine studies were included; seven were randomized controlled trials (RCTs), and two were observational studies. Three RCTs included were having a low risk of bias (ROB), two RCTs were having an unclear ROB, while two RCTs were having a high ROB. The nature of clinical pharmacist interventions included one or more components such as disease-related education, lifestyle changes, medication adherence counselling, medication therapy management, and discussions with physicians about prescription modification if necessary. Clinical pharmacist interventions reduce medication-related errors, improve therapeutic outcomes such as blood pressure, glycemic control, lipid control, CD4 T lymphocytes, and renal functions, and improve humanistic outcomes such as patient knowledge, adherence, and health-related quality of life. However, no study reported the economic outcomes of interventions.

**Conclusions:**

The findings of the studies included in this systematic review suggest that clinical pharmacists play important roles in improving patients’ health outcomes in Pakistan; however, it should be noted that the majority of the studies have a high risk of bias, and more research with appropriate study designs is needed.

**Supplementary Information:**

The online version contains supplementary material available at 10.1186/s12913-021-06897-0.

## Background

Since 1990, with pharmaceutical care introduction, pharmacists’ careers have evolved from single dispensary positions to patient-oriented health care [1, 2]. In developed countries, pharmacists are sufficiently trained to play a vital role in pharmaceutical care [3, 4]. However, in developing countries, pharmacists’ roles are gradually shifting toward ward rounds with other health professionals to monitor the patient’s progress, drug-related issues and communicate a medication therapy management plan [5–7]. Meanwhile, they continue to be primarily responsible for manufacturing, distributing, and dispensing medicines [3, 8]. Clinical pharmacists can offer patients a wide range of services, including prescription drugs and health-related services [9, 10]. They can assist physicians in prescribing drugs rationally, ensuring that patients understand the dosage regimen and method of administration, and improving patient adherence [11]. Moreover, pharmacists play an important role in public health promotion at community pharmacy settings, such as tobacco and alcohol control, nutrition and a healthy lifestyle, routine immunisation, infection prevention and treatment, and the management of mental health and other chronic disease care [12–14].

According to the World Health Organization’s (WHO) data repository on the Pakistan health force, the pharmacist ratio per 10,000 population in 2019 was 1.545 [15]. Currently, over 3000 pharmacists in Pakistan receive Doctor of Pharmacy (Pharm D) degrees each year from 21 public and 25 private universities [16]. Moreover, as of 2019, the number of community pharmacies in Pakistan has increased to more than 40,000 [17]. To improve the regulation of medicines across the country, the Federal Government of Pakistan has established a regulatory body, the drug regulatory authority of Pakistan (DRAP) Act 2012 [18, 19]. Under the Act, regulations range from existing basic services (i.e., dispensing, procurement, storage, distribution of therapeutic products and counselling) to enhanced medicine services (pharmaceutical care, pharmacovigilance, pharmacoepidemiology, pharmacoeconomic and services offered at drug information and poison centres) at all levels such as pharmacy, clinical, hospital, and community levels [19, 20]. In 2014, to strengthen pharmacists’ expertise in clinical roles, the higher education commission (HEC) introduces the Department of Pharmacy Practice in Pakistan’s private and public sector universities [21]. As a result, studies in Pakistan have begun to highlight potential clinical pharmacy progress, including further bedside activities, patient consultation, and therapy optimization in chronic conditions such as diabetes and hypertension [22–25].

Published literature reviews of clinical pharmacist interventions in the United States (US) and Western countries reported that different health care settings and disease management could benefit from clinical pharmacist care [26–30]. In 2013, Pande et al. carried out a systematic review of the impact of pharmacist interventions on patient outcomes, health service utilization, and costs in low-and middle-income countries (LMICs) [31]. The findings revealed that pharmacist services improve treatment outcomes such as hyperglycemia and systolic blood pressure (SBP), diastolic blood pressure (DBP), cholesterol control, and the quality of life of people living with chronic diseases such as asthma, diabetes, and hypertension [31]. However, the authors could not retrieve cost-related data, and the results were inconsistent because each study measured different outcomes with different clinical conditions using other measurement methods, necessitating careful interpretation. The review included all studies from middle-income countries such as southeast Asia, Africa, and Eastern Europe. As a result, the findings may not apply to countries with varying healthcare systems, such as Pakistan, an LMIC in southern Asia [18]. The utilization of clinical pharmacist services in Pakistan is not well established [32]. There may be a lack of awareness about the additional benefits of clinical pharmacist services and their potential implications in the Pakistani context, which could assist policymakers and stakeholders in using these services. Therefore, this systematic review aims to synthesize the therapeutic, safety, humanistic, and economic impact of clinical pharmacist interventions in Pakistani patients compared to standard treatments without the involvement of pharmacists in direct patient care.

## Methods

### Scope of review: eligibility criteria

This systematic review was conducted following the Cochrane Handbook for Systematic Reviews of the Intervention Guidelines [33], and the reporting followed the Preferred Reporting Items for Systematic Reviews and Meta-Analyses (PRISMA) Statement [34]. Studies were included if they were 1) randomized controlled trials (RCTs), non-RCTs (observational studies) such as pre-post without control group, follow up; 2) involved pharmacist intervention either alone or in a multidisciplinary team 3); measuring any health outcome (humanistic, safety, economic and therapeutic effects); 4) conducted among outpatients or inpatients in the hospital or community pharmacy settings; 5) had a control or comparison group (with healthcare professionals other than a pharmacist); 6) published in a peer-reviewed journal in English language and available in full-text.

### Information sources

We used a population, intervention, comparator, and outcome (PICO) search strategy in PubMed, Scopus, OVIDEmbase, CINAHL Plus, and Cochrane Libraries to find relevant records. The initial search was undertaken on 14th February 2021, with follow-up searches conducted on 28th February 2021.

### Database searching

From the database’s inception to 28th February 2021, a literature search was conducted using various search term combinations. The search terms used were (Pharmacist OR Pharmacy OR “Clinical Pharmacy” OR PharmD OR “Pharmacist-led”) AND (Adherence OR “Health outcomes” OR “Medication management” OR “Patient outcomes” OR outcome OR “Quality of life” OR “clinical outcome” OR Pharmacovigilance OR Economics OR “drug interactions” AND “drug safety”) AND (Pakistan OR Pakistani). Due to each database’s technical differences and limitations, the search mechanism in each database has been subsequently adapted and slightly modified (Supplementary file). Case reports, expert opinions, systematic reviews, letters to editors, comments, correspondences, news articles, qualitative studies, non-English studies, and conference abstracts were excluded if full articles were not available.

### Data screening and extraction

The author AA conducted the searches in relevent databases and were later independently reviewed by MS, MT, JD, AB, and SSH. All eligible studies were imported into the Endnote Version.X9.3.3 software (San Francisco, Clarivate Analytics) [35]. In the Endnote software, subgroups were created for each database. Endnote software was used to remove duplicates. The titles and abstracts were independently screened for inclusion in the full paper by all authors. AA performed a full paper screening using a preliminary screening form, and all authors independently reviewed it. The final inclusion of articles was based on mutual consensus. After selecting the eligible studies, the AA extracted the data independently using a standardized Cochrane data extraction form [36]. The extracted data were checked for accuracy and consistency by the second author (MS). Article details (objective, year of publication, and first authors), study design, country of study, sample size, and study characteristics (age, follow-up duration, pharmacist intervention, intervention strategy, control group, intervention group, type of outcome measure, all health outcomes) were extracted.

### Risk of Bias

Two reviewers (AA and MS) independently assessed the quality of RCTs using the Cochrane Risk of Bias Tool (ROB.2) [37]. Disagreements were resolved through mutual agreement. In non-RCTs, a Risk of Bias in non-Randomized Intervention Studies (ROBINS-I) tool was used for quality evaluation [38]. These studies have been assessed as being of low risk (if no bias), unclear risk (if any doubts affect results), and high risk (if bias has affected the results severely).

### Data synthesis

The findings of selected studies were qualitatively synthesized rather than combined for meta-analysis due to the authors’ high risk of bias judgments. This decision was made because the clinical and methodological approaches used in the studies differed. Using the extracted data, text summaries and summary tables were created.

## Results

### Study selection

**Fig. 1:**
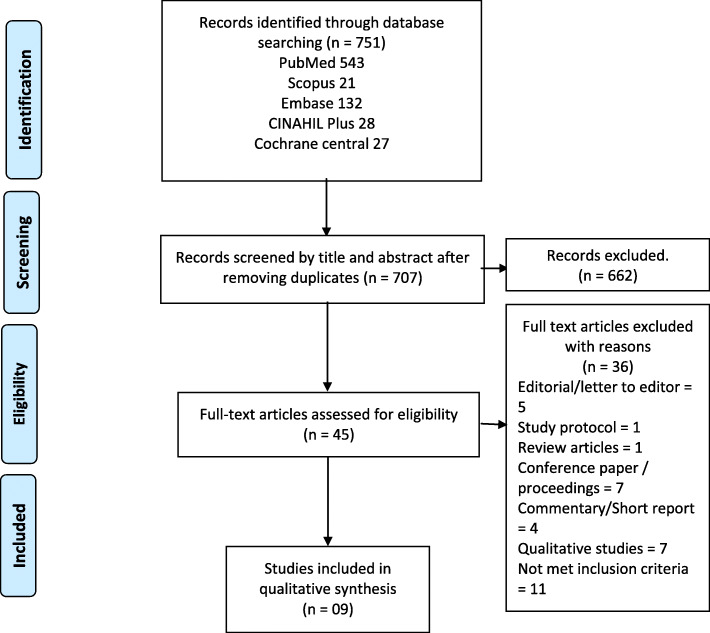
PRISMA flow diagram of included studies

The search strategy identified original research studies on the effect of clinical pharmacists’ interventions on therapeutic, safety, humanistic and cost-effective consequences of pharmacist intervention compared to usual care without pharmacist involvement in direct patient care in the Pakistani setting. Database searches yielded 751 papers. The use of EndNote software for de-duplication resulted in 707 papers being considered for preliminary screening by all authors for titles and abstracts. 45 papers were found to be eligible and underwent full paper screening. The bibliographies of the full-length articles were also reviewed, but no additional papers were discovered. Finally, nine studies were included in the qualitative synthesis. The search and screening processes are presented in a flowchart using a PRISMA diagram (Fig. 1) [34].

### Study characteristics

All of the studies were conducted between 2013 and 2020 and involved a total of 2931 patients. In eight studies, outpatients were enrolled [39–46], while inpatients were included in one study [47]. Patients with diabetes, hypertension, tuberculosis, chronic kidney disease (CKD), human immunodeficiency virus and hepatitis C infection were included in the studies. Of the nine studies, seven were randomized clinical trials (RCTs) [39–45], and two were observational studies (non-RCTs) [46, 47]. Therapeutic outcomes were studied in seven studies [39, 41–46], eight studies reported humanistic outcomes [39–46], and two studies discussed safety outcomes [43, 47]. None of the studies reported economic results. Pharmacist interventions were delivered (for example, at outpatient departments or inpatient departments), frequency of intervention range from 2 to 6 times during follow up (range 2 to 10 months), length of pharmacist intervention sessions (First session range 15 to 60 min, follow up sessions range from 10 to 45 min) reported in the studies.

### Risk of Bias

**Fig. 2:**
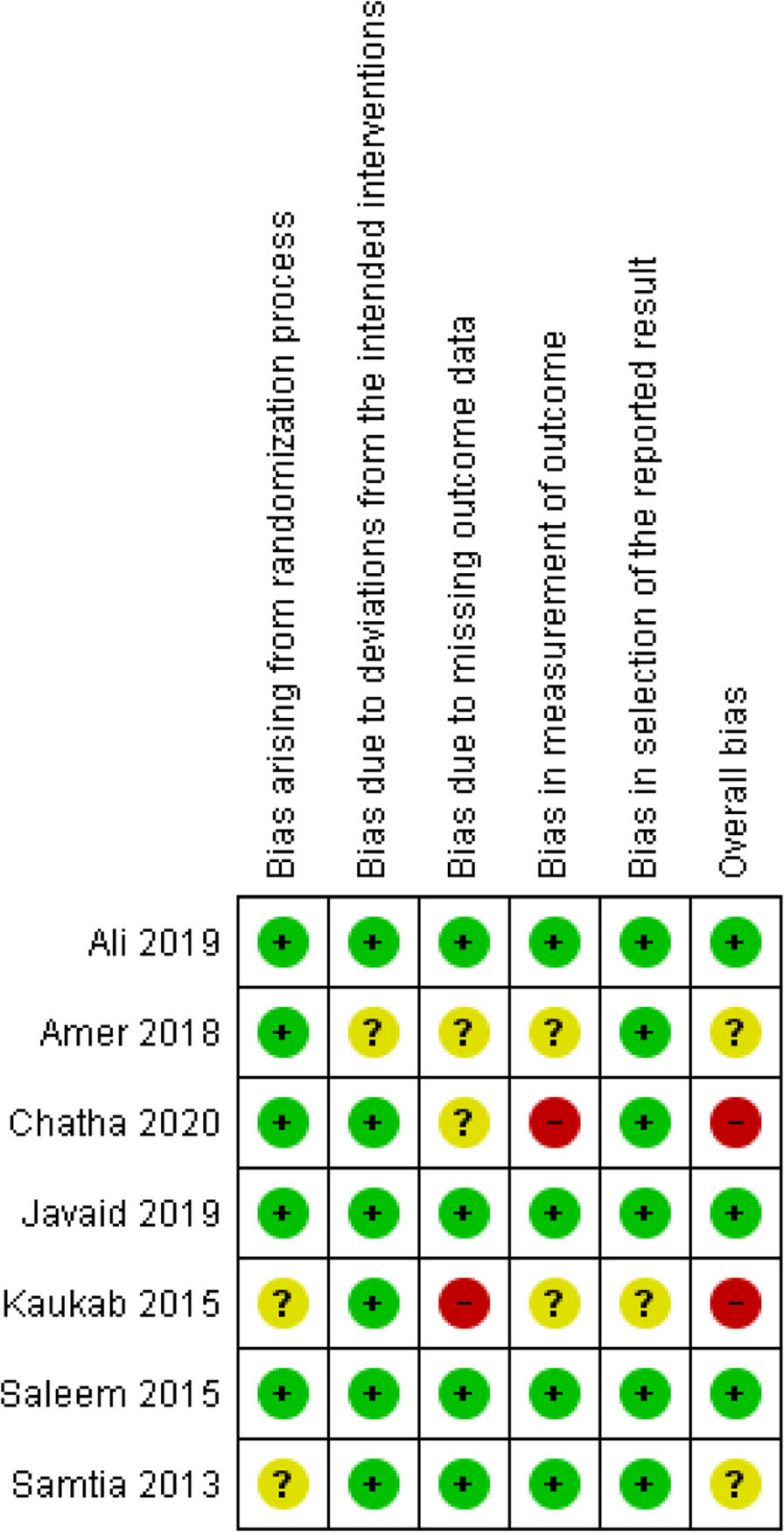
Risk of bias summary: review authors’ judgements about each risk of bias item for included RCTs

**Fig. 3:**
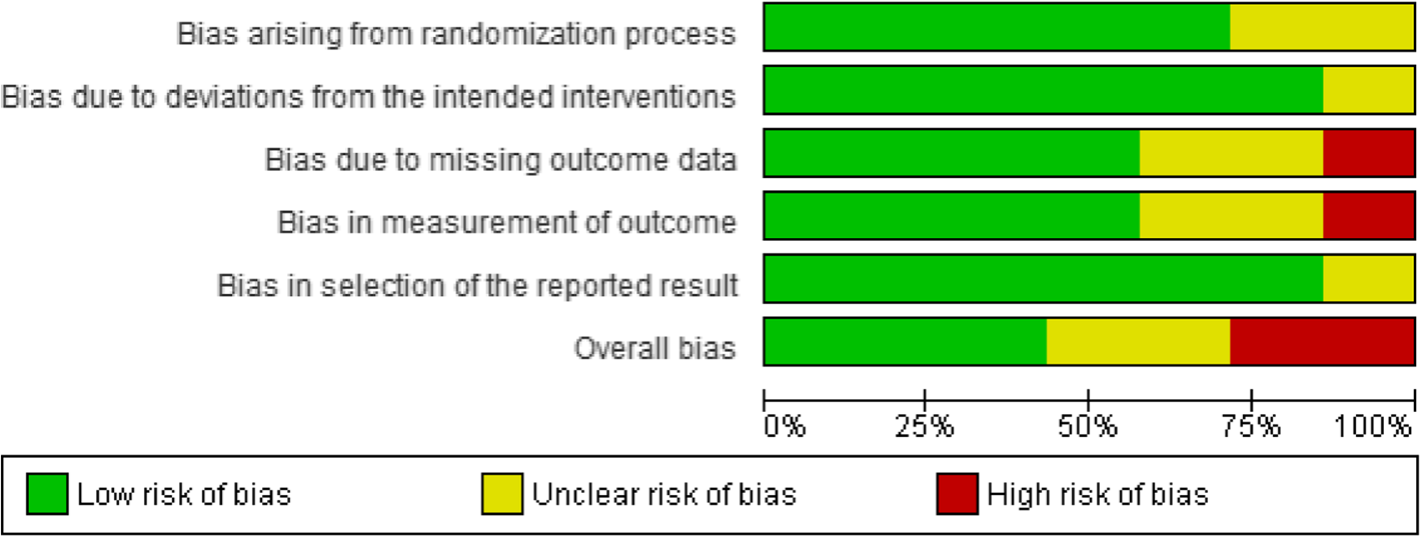
Risk of bias graph: review authors’ judgements about each risk of bias item presented as percentages across all included RCTs

**Fig. 4:**
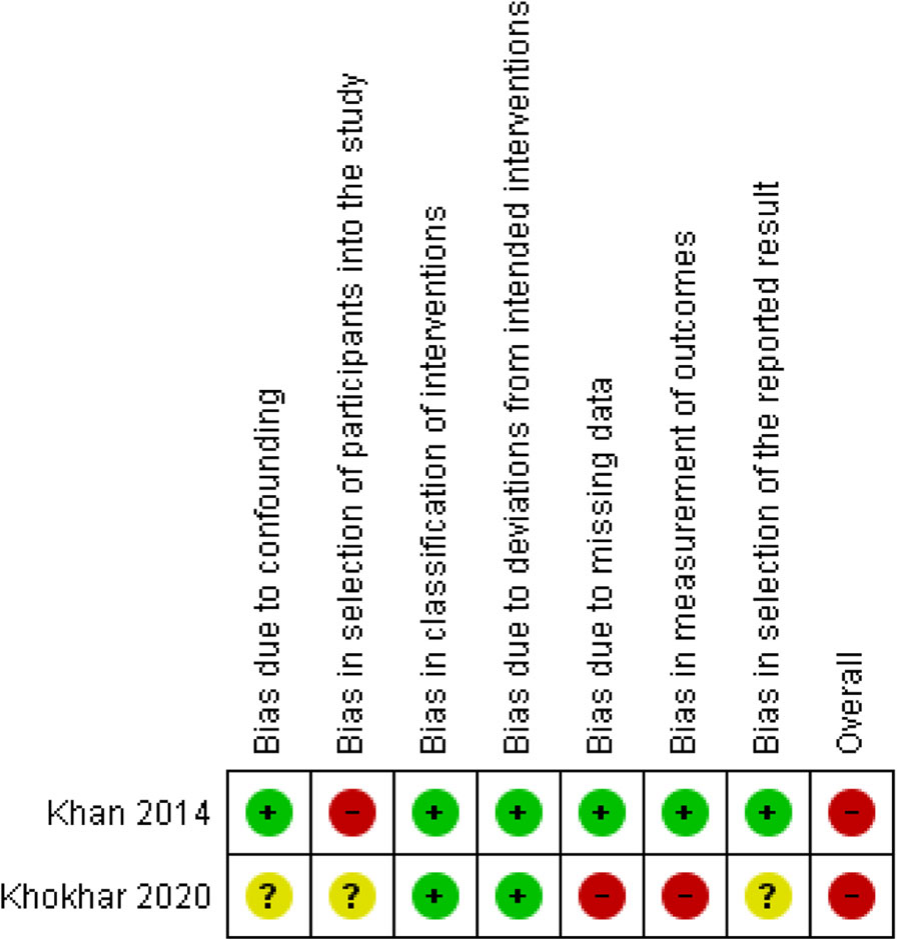
Risk of bias summary: review authors’ judgements about each risk of bias item for each included non-RCTs

**Fig. 5:**
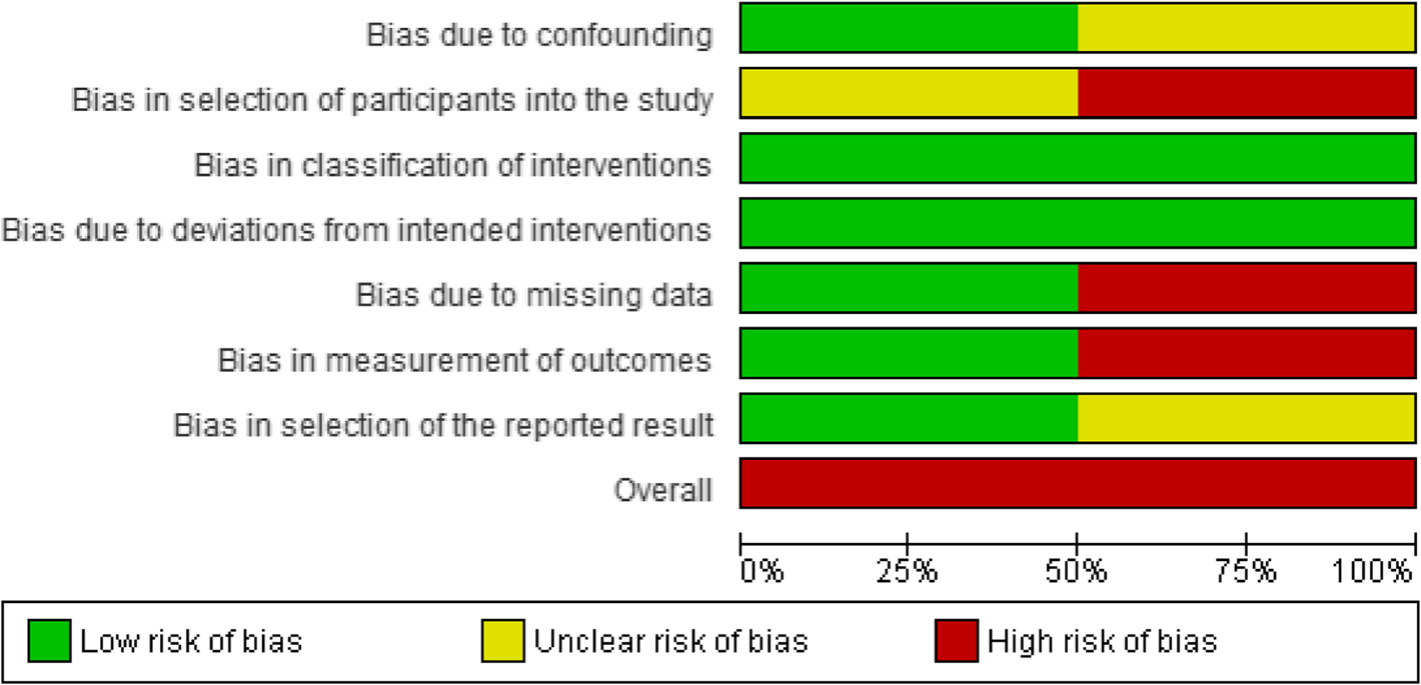
Risk of bias graph: review authors’ judgements about each risk of bias item presented as percentages across all included non-RCTs

Three RCTs included were having a low ROB [41, 43, 44], two RCTs were having an unclear ROB [39, 42], while two RCTs were having a high ROB [40, 45]. As part of the intervention, pharmacists were directly or indirectly involved in selecting participants and assessing outcomes in the majority of the RCTs in this review [39–43, 45]. Other common causes of bias included participant randomization issues, missing information of follow-up lengths, and handling missing data. Except for one study [41], none of the others provided pharmacists with training to help them deliver interventions. Both observational studies had a high ROB [46, 47]. Khan et al. failed to provide specific information about participants and the criteria used to purposefully sample participants, which introduces bias [47]. Khokhar et al. did not explain how outcome measurements were calculated or handled missing data [46]. Figures 2, 3, 4 and 5 show the review authors’ assessments of each risk of bias item for each included study, as well as the percentages of bias across all included studies for RCTs and non-RCTs separately.

### Nature of pharmacist interventions

Pharmacists provided a variety of interventions broadly classified into six categories 1) Provision of education regarding disease stages with booklets; 2) exploring adherence barriers and motivational interviewing to improve adherence; 3) lifestyle modification guidance; 4) pharmaceutical care consisting of pharmacovigilance, drug-drug interactions, drug-food interactions; 5) interacting with the physician to change the drug regimen 6) maintain patient follow up care.

Tables 1 and 2 summarised the study characteristics, patient outcomes, and the impact of pharmacist intervention on therapeutic, humanistic, and safety outcomes.

**Table 1:** Detailed characteristics of studies included in the review

Author (Year)	Objective	Study design	Sample size	Mean age Years	Follow up	Setting	Patients’ description	Pharmacist intervention	Outcomes			
									Thera	Hum	safety	eco
Samtia et al. 2013 [39]	Pharmacist led interventions on glycaemic control, medication adherence, disease knowledge, and lifestyle modifications among patients with diabetes	RCT	*N* = 348 IG = 178 CG = 170	IG = 46.1 CG = 42.3	5 months, follow up visit after every month	Outpatient settings of Nishtar Hospital Multan and DHQ Hospital Layyah	Diabetic patients who were receiving oral hypoglycaemic medication from at least the last 6 months and were having a BMI of more than 25 were included in this study. Patients solely on insulin therapy were not included.	Patients in the intervention group received pre-defined specialised care, i.e., education regarding the disease, adherence, dietary restrictions, self-monitoring of blood glucose, fasting blood glucose and guide patients about control of HBA1c, smoking cessation and exercise impact on glucose control. Also provide education regarding sensory changes, including foot examination	✓	✓	×	×
Khan et al. 2014 [47]	To check the impact of academic clinical pharmacists in reducing drug-related problems	prospective, observational, and interventional study	*N* = 373	NR	3 months	Inpatients of 250 bedded teaching-based hospital, located in Karachi	Inpatients of neonatal intensive care unit, Female surgical ward, male surgical ward, post-natal ward, cardiac ward, and medical ward.	Pharmacist on daily basis, monitored all aspects of patients’ drug therapy along with the past medical history, laboratory reports and practitioner’s notes. The identified drug-related problems were then discussed with the pharmacist team members, and with the development of consensus interventions were conveyed to the respective physician along with the best possible approach to rectify drug-related problem	×	×	✓	×
Kaukab et al. 2015 [40]	To analyse the effect of pharmacist individual counselling on depression in MDR-TB patients to improve depressive symptoms	RCT	*N* = 70 IG = 35 CG = 35	NR	10 months	TB department of Nishtar Hospital, Multan, Pakistan	Outpatients having drug resistance TB	Pharmacist provided educational intervention with economic help, e.g. monthly food basket (Ghee, flour, milk, sugar, tea, and all cereals). Two-way transportation fares free lab facilities, free medical check-up and free of cost medicines for whole month.	×	✓	×	×
Saleem et al., 2015 [41]	To assess the impact of an educational intervention provided to hypertensive through hospital pharmacists to improve their knowledge on hypertension, their adherence to the medication and their HRQoL	RCT	*N* = 385 IG = 193 CG = 192	39 ± 6.5	9 months, 3 follow up visits; first visit 15 min, later visits of 10 min	Cardiac units of SPH and BMCH located in Quetta	Outpatients of Patients aged 18 or over with an established medical diagnosis of hypertension, familiarity with Urdu (the national language of Pakistan) and on antihypertensive medication for the last 6 months.	Pharmacist provided health education about hypertension (nature, management, treatment and recommended diet and lifestyle modification), medication adherence and its importance in pharmacotherapy and HRQoL (conceptualisation and importance in treatment outcomes for hypertensive patients). The pharmacist also provided a pocket-sized educational book on hypertension, information leaflets and medication adherence cards (all in Urdu) during the counselling process	✓	✓	×	×
Amer et al. 2018 [42]	To evaluate the effect of pharmacist educational intervention to the patients of hypertension to improve knowledge, adherence to medicines, blood pressure control and HRQoL	RCT	*N* = 394 IG = 192 CG = 192	NA	9 months with 3 follow ups	Polyclinic hospital of Islamabad	Hypertension Outpatients visiting the cardiology section of the hospital	Pharmacist conducted interviews of patients at each visit and identified causes of lack to adherence towards medication and provided disease-related education to the patients (lifestyle education, medication counselling to increase knowledge about hypertension, adherence to medication and HRQol). A printed booklet (in Urdu language) of hypertension-related educational material was also provided to the patients.	✓	✓	×	×
Ali et al. 2019 [43]	To evaluate the impact of clinical pharmacy interventions on treatment outcomes, HRQoL, and medication adherence among hepatitis C patients.	RCT	*N* = 931 IG = 465 CG = 466	42.35	3 months, three follow up visits,	Gastroenterology outpatient department of SIMS, Lahore and the PIMS Islamabad	Confirmed HCV-positive patients aged ≥18 years who presented to the gastroenterology department during the study period were included.	Clinical pharmacist-provided individualised patient care, including direct patient monitoring, provision of medication diary, education on lifestyle modifications, and counselling on the appropriate use of HCV medication. Clinical pharmacy services continued until treatment completion.	✓	✓	✓	×
Javaid et al. 2019 [44]	To demonstrate the pharmacist-led improvements in glycaemic, blood pressure and lipid controls in type 2 diabetes mellitus (T2DM) patients	RCT	*N* = 244 IG = 123 CG = 121	50.92	9 months with 3 follow-ups; 15–30 min	primary care facility, Murad clinic Shalamar link road, Lahore,	Un-controlled T2DM patients (HbA1c > 8%) were included in study.	Pharmacist performed PWDT, CORE, PRIME, non-adherence, adverse drug reactions, monitoring and screening of patients at each follow-up.	✓	✓	✓	×
Chatha et al. 2020 [45]	To investigate pharmacist-led interventions to improve adherence to antiretroviral therapy (ART) for people living with HIV	RCT	*N* = 66 IG = 33 CG = 33	IG = 36. 18 CG = 31.39	2 months, two follow ups of 30 min duration	Antiretroviral therapy centre (ART), Pakistan institute of medical sciences	HIV positive, > 18 age, taking ART for > 3 months. Patients were exluded if having incomplete baseline blood tests, pregnancy, or a cognitive impairments.	Pharmacist provided counselling was tailored to each social factor focused on personal barriers to taking medication and was aimed at helping participants understand their medication-taking behaviours while acknowledging the actions needed to maintain a high level of adherence, also included advice on the potential negative impact of diet and supplementary herbs or medicines on the effectiveness of ART	✓	✓	×	×
Khokhar et al. 2020 [46]	To evaluate the impact of pharmacist-led intervention among pre-dialysis CKD patients to improve disease knowledge, adherence, body composition and physiological profile of CKD patients.	Pre-post prospective	*N* = 120 IG = 60 CG = 60	55.88 ± 13.83	3 months; 45 min first session	Nephrology outpatient departmen, National Institute of Kidney Diseases, Sheikh Zayed Hospital, Lahore	All patients with an established diagnosis of CKD stage 2 to 4 (GFR between 15 and 89 ml/min per 1.73 m^2^) according to KDOQI guidelines of the National Kidney Foundation were enrolled in the study.	Pharmacist provided information about the disease, dietary recommendations, counselling to improve medication adherence along with telephonic follow-up	✓	✓	×	×

Thera = Therapeutics, Hum = Humanistic, Eco = Economic, FIVs = Follow on interventions, DHQ = District headquarter hospital, BMCH = Bolan Medical Complex Hospital, BMI = Body mass index, MDR-TB = Multidrug-resistant tuberculosis RIVs = rejected interventions, HRQoL = Health Related Quality of Life, SPH = Sandeman Provincial Hospital, SIMS = Services, Institute of Medical Sciences, PIMS = Pakistan Institute of Medical Sciences, PWDT = Pharmacist’s work up of drug therapy, CORE = Condition, Outcome, Regime, Evaluation, PRIME = Problem, Risk, Interaction, Mismatch, Efficacy, CKD = chronic kidney disease, KDOQI = Kidney Disease Outcomes Quality Initiative, GFR = Glomerular filtration rate

**Table 2:** Summary of the clinical pharmacist effect on patients’ outcomes

Authors	Therapeutic	Safety	Humanistic	Economic
Samtia 2013 [39]	FBS* HBA1C* Waist Circumference*	–	Compliance (+)	–
Khan et al. 2014 [47]	–	DRP	–	–
Kaukab et al. [40]		–	Depression (+)	–
Saleem et al., 2015 [41]	SBP (+) DBP (+)	–	Knowledge (+) Adherence (+) EQ 5D (−) EQ-VAS (+)	–
Amer et al. 2018 [42]	SBP (+) DBP (+)	–	Knowledge (+) Adherence (+) EQ 5D (+) EQ-VAS (+)	–
Ali et al. 2019 [43]	SVR 12 (+)	ADE (+) DDI (+)	Adherence (+)	–
Javaid et al. 2019 [44]	Waist* BMI (+) HbA1C (+) SBP (+) DBP (+) Cholesterol (+) Triglycerides (+) Serum creatinine (+) eGFR (+)	ADE (+) DDI (+)	Knowledge (+)	–
Chatha et al. 2020 [45]	CD4 Cell Count (+)	–	Adherence (+)	–
Khokhar et al. 2020 [46]	CBC* RFT* Blood Glucose* Electrolytes*	–	Knowledge (+) Adherence (+)	–

*No significant (*P* > 0.05) difference between intervention and control group, + = significant (*P* < 0.05) effect in favor of intervention group, − = significant (*P* < 0.05) effect in favor of control group SVR 12 = sustained virological response at 12 weeks, FBS = Fasting blood sugar, ADE = adverse drug event, CBC=Complete Blood count, RFT = renal function test, BMI = Body mass index, SBP = Systolic blood pressure, DBP = Diastolic blood pressure, eGFR = estimated glomerular filtration rate

### Impact of pharmacist interventions on therapeutic outcomes

Three studies reported clinical pharmacist interventions significantly reduced the SBP and DBP in hypertension patients [41, 42, 44]. Saleem et al. detected significant reduction in mean SBP (mean difference: IG = 8.4 vs CG = 0.2; *p* = 0.004) and DBP (mean difference: IG = 6.6 vs.CG = 0.4; *p* = 0.009) in intervention group compared to control group [41]. Amer et al. also reported that pharmacist-led intervention significantly improved hypertension as SBP (IG:131.81 vs. CG:137.91) and DBP (IG:83.75 vs. CG:87.77) was considerably lower in the intervention group compared to the control group (*p* < 0.001) [42]. Similarly Javaid et al. reported that participants in intervention arm had better improvement in SBP (mean difference = IG: − 21.1 vs. CG: + 6.1; *p* < 0.001) and DBP (mean difference = IG: − 7 vs. CG: + 4; *p* < 0.001) than control arm [44].

Three studies on the impact of clinical pharmacist’s interventions in diabetes care were published, with findings ranging from positive to significant [39, 44, 46]. Samtia et al. reported that there was no statistical difference in mean fasting blood glucose (mean difference: -11.95; *P* = 0.116) and HbA1C level (mean difference: -0.43; *P* = 0.112) between the intervention group and control group at five months follow-up [39]. Kokhar et al. also reported similar findings as there was no significant change in fasting and random blood glucose level at baseline and follow-up [46]. On the contrary to these findings, Javaid et al. reported that at follow-up, participants in the intervention arm 10.9 ± 1.7 vs. 7.7 ± 0.9) had significantly better improvement in HbA1C level compared to the control arm (10.3 ± 1.3 vs. 9.7 ± 1.3) [*p* < 0.001] [44].

Samtia et al. reported that pharmacist-led intervention had significantly reduced body mass index (BMI) (mean difference: − 1.87; *p* = 0.014) and waist circumference (mean difference: − 1.27; *p* = 0.002) of diabetic patients in the intervention group [39]. Chatha et al. reported that at the end of the follow-up period, the intervention group had statistically significant increases in CD4 lymphocytes cells compared to the usual care group (*p* = 0.005) [45]. Similarly, Javaid et al. reported that for various process outcome measures, inter-group improvements were more significant in the intervention group at final follow up in comparison to the control group; SBP (*p* < 0.0001), DBP (*p* = 0.02), cholesterol (*p* < 0.0001), triglycerides (*p* < 0.0001), serum creatinine (*p* < 0.001), estimated glomerular filtration (eGFR) (*p* < 0.001).

### Impact of pharmacist interventions on humanistic outcomes

Samtia et al. reported that the pharmacist intervention group had shown improved adherence (*p* = 0.003), improved knowledge regarding sensory changes (*p* < 0.001), self-monitoring of blood glucose level (*p* = 0.001), and knowledge regarding exercise (*p* < 0.001) compared to the control group [39]. Saleem et al. observed at follow-up there was a significant improvement in adherence (− 1.8 vs. 3.2; *p* < 0.001) and disease-related knowledge (7.5 vs. 10.2; *p* < 0.001) among participants who received pharmacist intervention [41]. Similar results were reported by Amer et al. that group that received the pharmacist intervention had improved adherence (IG: 5.89 vs. CG:3.89; *p* < 0.001) and disease-related knowledge score (IG: 18.18 vs. CG:13.31; *p* < 0.001) compared to patients in the control group [42]. Likewise, Ali et al. revealed that hepatitis C patients in the pharmaceutical care group had better (88.6%) adherence than patients in the usual care group (77.9%) (p < 0.001) [43]. Chatha et al. also observed that educational intervention significantly improved the medication adherence among HIV patients as a proportion of patients who never missed their medication was increased up to 36% in the intervention group compared to only a 3% change in the usual care group [45]. Kokhar et al. evaluated the medication adherence and knowledge scores among CKD patients. At follow-up, a significant improvement was observed in medication adherence (*p* = 0.042) and knowledge scores (*p* = 0.022) of participants in the intervention group compared to the control group [46]. Also, Kaukab et al. studied the impact of pharmacist education and socioeconomic support on the depression status among drug-resistant tuberculosis patients. At ten months follow-up, patients who received education and support had significant improvement in depression symptoms than the control group [31].

Amer et al. reported that after the pharmacist intervention, the participants had significantly improved health-related quality of life (HRQoL) score (IG: 0.73 vs. CG: 0.68; *p* < 0.001) and VAS score (IG: 69.43 vs. CG: 64.29; p < 0.001) compared to the control group [42]. Ali et al. reported that HRQoL was significantly improved in both the usual care and pharmaceutical care groups, but no statistically significant change was observed between them. While there was a significant difference in visual analog scale (VAS) score between both groups at follow-up as patients in the pharmaceutical care group had higher scores than the usual care group (*p* < 0.001) [43]. Interestingly Saleem et al. reported that at follow up the quality of life was significantly reduced (42.2 vs. 39.6; *p* < 0.001) in the intervention group [41].

### Impact of pharmacist interventions on safety outcomes

Pharmacists actively provided pharmaceutical care, identified drug-related issues, and reported them to physicians to change prescriptions [43, 44, 47]. For example, Khan et al. reported that clinical pharmacists investigated the 373 inpatients profiles and identified 147 drug-related problems (DRP), of which 41.5% (*n* = 61) were related to adverse drug reactions. To solve these problems, 161 recommendations like the change of drug, dosage adjustments were made by a clinical pharmacist, of which 139 (86.33%) successfully solved the issues [47]. In addition, Ali et al. evaluated the frequency of adverse drug events and reported that fewer patients in the pharmaceutical care group (8.2%) had experienced adverse drug events than the usual care group (10.5%) [43].

## Discussion

To the best of the authors’ knowledge, this is the first systematic review to include widespread evidence of clinical pharmacists’ role in Southern Asia, particularly in an LMIC like Pakistan. This systematic review incorporates evidence from nine studies in which the primary intervention provided by clinical pharmacists was disease-specific education, followed by motivational interviewing of patients to improve treatment adherence and medication therapy management to improve patients’ health outcomes. All studies found that clinical pharmacist interventions improved therapeutic outcomes (SBP, DBP, HBAIc, Blood glucose, CD-4 T lymphocytes, serum creatinine levels, eGFR) and safety outcomes (drug-related problems like drug-drug interactions). Interventions also improved humanistic outcomes such as disease knowledge, treatment adherence, depression, and HRQoL in all studies except Saleem et al [41], where HRQoL of the intervention group was surprisingly got lower, maybe due to comorbidities or higher depression scores in chronic disease patients due to associated psychological distress [48, 49]. In Pakistan, clinical pharmacy education is evolving, but it is still at its foundational level [32]. Despite widespread recognition of the need for advanced pharmacy education, clinical pharmacist capacity and experience are severely lacking in LMICs [50]. LMICs must develop a mandatory continuing professional development (CPD) model for clinical pharmacists to update, advance, and update their training and skills in this context [51]. Furthermore, CPD in LMICs should strengthen the pharmacy system and its role in improving clinical pharmacy practise [50]. The review findings may persuade policymakers in Pakistan that clinical pharmacists can improve patients’ health outcomes and healthcare systems. Furthermore, stakeholders can benefit from Babar’s ten recommendations for advancing pharmacy practise in Pakistan [8].

The provision of simple education (about the disease, therapy, lifestyle, potential consequences of lack of adherence) was the most common intervention by the clinical pharmacist. Few studies evaluated complete pharmaceutical care follow-up, including optimizing medication therapy, monitoring disease progression, assessing adherence, identifying and resolving drug-related problems by communicating with physicians, and maintaining manual records for each patient [41, 43, 44, 46, 52]. This demonstrates that identifying drug-related problems (DRPs) through a pharmacist review can improve patient medication safety, as other studies have also shown [53–55]. Clinical Pharmacists are primarily concerned with DRPs. These issues must be identified and resolved to achieve their therapeutic goals and achieve the best possible outcomes from their drug therapy. Given the high number of DRPs reported by studies in Pakistan [56–61], clinical pharmacists in Pakistan have an excellent opportunity to resolve these issues and improve patients’ health outcomes. We found cross-sectional [62, 63] and qualitative studies [64, 65] from community pharmacy settings, but we couldn’t find any follow-up studies from community pharmacy settings, so we recommend further development of pharmacist activities at community pharmacies as these provide an alternative for the public to obtain medicines and access to basic, minor health-related services.

The studies included in this review ranged in quality, had methodological heterogeneity, versatility in outcome measurement, and reported on selected outcomes with varying pharmacist interventions. Regarding pre-training of clinical pharmacists about the intervention, only one study provided data [41]. Nonetheless, clinical pharmacists played an important role in identifying and addressing therapy-related issues in chronic diseases (diabetes, Hepatitis C, CKD, hypertension, tuberculosis, and HIV). These findings are comparable with the study conducted in a Jordanian upper-middle-income setting [3]. However, we could not find any research that evaluated the cost-effectiveness of pharmacist intervention. Similarly, a Cochrane review also reported limited evidence of the cost of pharmacist interventions in LMICs [31]. In terms of safety outcomes, the review found little evidence of clinical pharmacist intervention; similarly, less evidence of safety was generated and reported from the United States of America (USA) and European countries [13, 27, 66, 67]. Involving the clinical pharmacist might come based on task shifting by the physicians towards clinical pharmacists to take the responsibility of therapeutic medication management, but still, it relies on the credibility, confidence, and trust, which may achieve with meeting therapy goals.

### Implications for practice and research

Clinical pharmacist roles are emerging, and this review highlighted the impact of these services in the Pakistani context. However, acceptance of their clinical roles by other healthcare workers is sometimes challenging [20, 32]. The barriers to engaging pharmacists in collaborative care could be overcome by building trust and demonstrating the value of pharmacists in health care teams and strategically engaging stakeholders, including legal departments, in the development of the collaborative practise process. Moreover, there should be multidisciplinary group discussions to advance clinical pharmacy services in Pakistan. Only Saleem et al. reported on the type of training given to pharmacists prior to implementing the research intervention [41]. Disease epidemiology, treatment, prevention, pharmacotherapy, strategies to overcome adherence barriers, the importance of treatment outcome, health education, effective communication skills, patient counselling techniques, and the importance of HRQoL assessment in treatment outcome assessment were all covered in the pharmacist training. Each year, many pharmacists are produced in Pakistan; however, the problem is with their clinical practise training [68]. Students in their final years have some fix visits to hospitals, but they are not given enough time or training during graduation to become experts in clinical settings. No professional body in Pakistan certifies pharmacist specialties, such as the board of pharmacy specialties (BPS) in America, which certifies pharmacists in specialised services [69]. As of August 2021, only twelve BPS certified pharmacists are working in different hospitals of Pakistan [70]. Government (Govt) of Pakistan should start initiatives like forming a council at a state level to begin clinical residency and certification programs to strengthen pharmacists to take better responsibility for patients’ pharmaceutical care. Moreover, govt should start continuous education programs like in the United Kingdom (UK) 30 h of ongoing professional development are necessary to complete per year [71].

Future research should focus on the safety and cost-effectiveness of clinical pharmacist interventions to further develop pharmacist roles. Adequately powered randomised studies with standardised outcome measurements, longer intervention duration, and equal baseline between groups will be required in the future. Research is also needed on pharmacist interventions’ time, frequency, and content to improve clinical outcomes [72]. Furthermore, this study concludes essential insight for future research focusing on a tailored intervention and the cost of delivering future cost-effective interventions. The result will be beneficial for the policymakers to choose pharmacist interventions based on the availability of their resources.

### Strengths and limitations

We have previously seen clinical pharmacist reviews from developed or upper-middle-income countries, but there is no review from LMIC. This review focuses on an LMIC where the clinical pharmacy is still in its early stages of development. Evidence suggests that clinical pharmacists’ participation in the healthcare team improves patients’ health outcomes. Our findings support the provision of more clinical residency training to pharmacy graduates, who can play a more important role in improving patient health outcomes and cost savings for the health care system and society.

There are some limitations of this review. First, to avoid bias, only peer-reviewed published studies were included in this review; unpublished studies were not included. Second, we found one or a maximum of two studies for each outcome, so it was practically impossible to apply meta-analysis due to follow-up variation, high risk of bias, and intervention content differences. Third, there was variation in health outcome measurements as well as heterogeneity in pharmacist interventions. Fourth, only evidence from Pakistan was included; data from neighbouring countries were not included due to different healthcare systems. Despite limitations, this review can help to advance clinical pharmacy development in LMICs and thus improve patient outcomes.

## Conclusion

The review underlined the role of the clinical pharmacist services in improving patient outcomes and medication therapy management. Clinical pharmacist interventions showed a positive impact on therapeutic, humanistic, and safety outcomes. However, much remains to be understood in cost, and long-term intervention impact. Future studies must be more rigorous in terms of evaluating multidimensional and long-term outcomes. Evidence of Costs-effectiveness must also be sought to allow informed decision-making and allocation of resources. The findings of this review will be of interest to policymakers, particularly in areas where new clinical pharmacy services are being developed.

## Supplementary Information

**Additional file 1.** Search strategies.

## Data Availability

All data is presented within the manuscript.

## Abbreviations

DRAP: Drug Regulatory Authority of Pakistan
HEC: Higher Education Commission
PRISMA: Preferred Reporting Items for Systematic Reviews and Meta-Analyses
PICO: Population, intervention, comparator, and outcome
ROB: Risk of Bias
ROBINS: Risk of Bias in non-Randomized Intervention Studies
RCTs: Randomized clinical trials
SBP: Systolic blood pressure
DSP: Diastolic blood pressure
HRQoL: Health related quality of life
LMICs: lower-middle-income countries
CPD: Continuing professional development
DRP: Drug-related problems
