# Supplementary material for: The impact of clinical pharmacist services on patient health outcomes in Pakistan: a systematic review
Source: BMC Health Serv Res. 2021 Aug 23;21:859. doi: 10.1186/s12913-021-06897-0 (PMC8381566; doi:10.1186/s12913-021-06897-0)
Supplement: Supplementary file 1 — Additional file 1. Search strategies. [file 12913_2021_6897_MOESM1_ESM.docx]

**Search strategies**

**PubMed**

1. Pharmacist OR Pharmacy OR “Clinical Pharmacy” OR PharmD OR “Pharmacist-led”

2. Adherence OR “Health outcomes” OR “Medication management” OR “Patient outcomes” OR outcome OR “Quality of life” OR “clinical outcome” OR Pharmacovigilance OR Economics OR “drug interactions” OR “drug safety”

3. Pakistan OR Pakistani

4. 1 AND 2 AND 3 (Upto 28^th^ February 2021)

**Cinahl** (Upto 28th February 2021)

1. Pharmacist.mp. or community pharmacist/ or clinical pharmacist/ or pharmacist/ or hospital pharmacist/ or pharmacist patient relationship/
2. medication management.mp. or medication therapy management/
3. adherence.mp. or Morisky Medication Adherence Scale/ or medication adherence monitoring system/
4. medical service/ or patient/ or pharmaceutical care/ or therapy/
5. outcome assessment/
6. economics/ or health economics/
7. "quality of life"/th [Therapy]
8. pharmacovigilance/ or drug surveillance program/ or drug safety/
9. pakistan.mp. or Pakistan/
10. 2 OR 3 OR 4 OR 5 OR 6 OR 7 OR 8
11. 1 AND 9 AND 10

**Scopus**

TITLE-ABS-KEY ( pharmacist OR pharmacy OR “clinical AND pharmacy” OR pharmd OR “pharmacist-led” ) ) AND ( TITLE-ABS-KEY ( adherence OR “health AND outcomes” OR “medication AND management” OR “patient AND outcomes” OR outcome OR “quality AND of AND life” OR “clinical AND outcome” ) ) AND ( TITLE-ABS-KEY ( pakistan OR pakistani ) )

(Upto 28^th^ February 2021)

**Cochrane library** (Upto 28^th^ February 2021)

1. Pharmacist.mp. or community pharmacist/ or clinical pharmacist/ or pharmacist/ or hospital pharmacist/ or pharmacist patient relationship/
2. medication management.mp. or medication therapy management/
3. adherence.mp. or Morisky Medication Adherence Scale/ or medication adherence monitoring system/
4. medical service/ or patient/ or pharmaceutical care/ or therapy/
5. outcome assessment/
6. economics/ or health economics/
7. "quality of life"/th [Therapy]
8. pharmacovigilance/ or drug surveillance program/ or drug safety/
9. pakistan.mp. or Pakistan/
10. 2 OR 3 OR 4 OR 5 OR 6 OR 7 OR 8
11. 1 AND 9 AND 10

**OVIDEmBase** (Upto 28^th^ February 2021)

1. Pharmacist.mp. or community pharmacist/ or clinical pharmacist/ or pharmacist/ or hospital pharmacist/ or pharmacist patient relationship/
2. medication management.mp. or medication therapy management/
3. adherence.mp. or Morisky Medication Adherence Scale/ or medication adherence monitoring system/
4. medical service/ or patient/ or pharmaceutical care/ or therapy/
5. outcome assessment/
6. economics/ or health economics/
7. "quality of life"/th [Therapy]
8. pharmacovigilance/ or drug surveillance program/ or drug safety/
9. pakistan.mp. or Pakistan/
10. 2 OR 3 OR 4 OR 5 OR 6 OR 7 OR 8
11. 1 AND 9 AND 10
